# Validated Microsurgical Training Programmes: A Systematic Review of the Current Literature

**DOI:** 10.3390/jcm14217452

**Published:** 2025-10-22

**Authors:** Victor Esanu, Teona Z. Carciumaru, Alexandru Ilie-Ene, Alexandra I. Stoia, George Dindelegan, Clemens M. F. Dirven, Torstein Meling, Dalibor Vasilic, Victor Volovici

**Affiliations:** 1 Center for Complex Microvascular Surgery, Erasmus MC, 3015 GD Rotterdam, The Netherlands; v.esanu@erasmusmc.nl; 2 Department of Experimental Microsurgery, Simulation and Experiment Center, Iuliu Hațieganu University of Medicine and Pharmacy, 400347 Cluj-Napoca, Romania; ioana.alex.stoia@elearn.umfcluj.ro; 3 Department of Plastic and Reconstructive Surgery, Erasmus MC, 3015 GD Rotterdam, The Netherlands; t.carciumaru@erasmusmc.nl (T.Z.C.); d.vasilic@erasmusmc.nl (D.V.); 4 Department of General Surgery, Iuliu Hațieganu University of Medicine and Pharmacy, 400347 Cluj-Napoca, Romania; ilieene.alexandru@gmail.com; 5 Department of Neurosurgery, Erasmus MC, 3015 GD Rotterdam, The Netherlands; c.dirven@erasmusmc.nl; 6 Department of Neurosurgery, Rigshospitalet, 2100 Copenhagen, Denmark; torstein.ragnar.meling@regionh.dk

**Keywords:** microsurgery, training, validation, programme, predictive validity, skill acquisition, skill retention

## Abstract

**Background:** Microsurgical skill acquisition and development are complex processes, due to the often complex learning curve, limited training possibilities, and growing restrictions on working hours. Simulation-based training programmes, employing various models, have been proposed. Nevertheless, the extent to which these training programmes are supported by scientific evidence is unclear. The aim of this systematic review is to evaluate the extent and quality of the scientific evidence backing validated microsurgical training programmes. **Methods:** A systematic literature review was conducted, following a study protocol established a priori and in accordance with the PRISMA guidelines. The databases searched were the Web of Science Core Collection (Web of Knowledge), Medline (Ovid), Embase (Embase.com), and ERIC (Ovid). Studies were included if they described microsurgical training programmes and presented a form of validation of training effectiveness. Data extraction included the number of participants, training duration and frequency, validation type, assessment methods, outcomes, study limitations, and a detailed training regimen. The risk of bias and quality were assessed using the Medical Education Research Study Quality Instrument (MERSQI). Validity was assessed using an established validity framework (content, face, construct, and criterion encompassing both concurrent and predictive validity). The Level of Evidence (LoE) and Recommendation (LoR) were evaluated using the Oxford Centre for Evidence-Based Medicine (OCEBM). **Results:** A total of 25 studies met the inclusion criteria. Training programmes were classified into one-time intensive courses or longitudinal curricula. Face, content, and construct validity were the most commonly assessed aspects, while predictive validity was the least frequently assessed and not properly evaluated. Training models ranged from low-fidelity (silicone tubes, synthetic vessels) to high-fidelity (live animal models). The Global Rating Scale (GRS), the Structured Assessment of Microsurgery Skills (SAMS), and the Objective Structured Assessment of Technical Skills (OSATS) were the most frequently used objective assessment tools for evaluation methods within the programmes. The risk of bias MERSQI score was 12.96, ranging from 10.5 to 15.5, and LoE and LoR scores were moderated. Across the studies, 96% reported significant improvement in microsurgical skills among participants. However, most studies were limited by small sample sizes, heterogeneity in baseline skills, and a lack of long-term follow-up. **Conclusions:** While validated microsurgical training programmes improve skill acquisition, challenges remain in terms of standardisation and best cost-effective methods. Future research should prioritise evaluating predictive validity, creating standardised objective assessment tools, and focus on skill maintenance.

## 1. Introduction

Microsurgical skill acquisition poses unique challenges due to the complex learning curve [1], the highly technical nature of the procedures, and the limited accessibility of microsurgical training centres [2]. Taking into account working hour regulations [3], the increased emphasis on patient safety, and the growing administrative burden, dedicated training remains difficult to prioritise. Consequently, traditional models, such as the Halstedian “see one, do one, teach one” approach [4,5], are becoming less feasible in the current clinical landscape. There is a need to define which training programmes achieve the fastest, most effective means of helping participants overcome the challenges of the learning curve, while also improving patient outcomes and reducing costs.

Besides the technical aspect, cognitive abilities such as spatial awareness and fine motor coordination play an important role in training [6]. Despite substantial advancements in the techniques and tools employed in training laboratories, such as the incorporation of novel low- and moderate-fidelity models [7,8,9,10,11], virtual reality simulations [12,13,14] or even hybrid approaches like online web-based curricula [15], microsurgical training requires robust, validated training programmes backed by solid scientific evidence [16], in order to address the demands and complexities of current surgical practice.

These constraints have allowed for a paradigm shift towards simulation-based training models, enabling skill acquisition and refinement in a relatively risk-free environment [17]. Although many training models and programmes are described in the literature, few are adequately validated [18]. Furthermore, the diversity of models and their associated costs represent a major drawback to widespread implementation.

Validated training programmes constitute the key for effective, comprehensive microsurgical education, as well as for addressing disparities in quality of microsurgical care and access to training resources [18]. The aim of this systematic review is to evaluate the current literature on validated microsurgical training programmes, to determine whether validated microsurgical training programmes improve objective technical performance among medical students, residents, and surgeons compared with baseline or alternative training, and to summarise evidence on predictive validity, skill retention, model fidelity, and resource use, seeking to identify the best practices and highlight areas for further research.

## 2. Materials and Methods

A systematic review was carried out following the Preferred Reporting Items for Systematic Reviews and Meta-Analyses (PRISMA) 2020 guidelines. The study was not registered in advance, thus the methodology was determined a priori and documented in a protocol, available in Supplementary Materials S1.

### 2.1. Search Strategy

The search strategy was developed and conducted in collaboration with the academic librarian at Erasmus MC Medical Center. The databases searched included Web of Science Core Collection (Web of Knowledge), Medline (Ovid), Embase (Embase.com), and ERIC (Ovid). The searches covered the period from database inception to 27th January 2025. The search strings were made by combining terms associated with “microsurgery,” “training”, “validation”, “programs,” and optimisation of the work strategy (Supplementary Materials S2).

### 2.2. Selection and Eligibility Criteria (PICOS)

Screening was conducted using Covidence software (Veritas Health Innovation, Melbourne, Australia), with all articles independently reviewed by title and abstract by two authors (VE and AIE). Duplicates were removed using Covidence’s deduplication feature with manual verification. Conflicts were discussed and resolved with the senior author (VV). English-language studies focusing on microsurgical training programmes that provided a form of validation and objectively measured their effectiveness through validated endpoints, such as performing a microvascular anastomosis in students/residents/surgeons with baseline/alternative/none as comparators, using RCT, non-randomised comparative or before-and-after designs reporting objective performance (primary) and/or patency/time, predictive validity, skill retention, model fidelity, and resources (secondary) were included. Non-English papers, non-validated training programmes, and studies involving other training regimens where the endpoint was not a microvascular anastomosis were excluded. Disputes were settled by consensus or by consulting the senior author. Full-text reviews were performed for articles that passed the title/abstract sifting phase by two authors (VE and TZC).

### 2.3. Data Extraction and Analysis

A standardised data extraction form was used to collect relevant information from all the articles included in the full-text review by two reviewers independently (VE and TZC). Discrepancies were resolved by discussion. This contained information regarding title, author, year of publication, the Detailed Training Programme described in the paper, the number, type, and baseline skill level of participants, the duration and frequency of the training regimen, the validation type, assessment methods used, and the main outcomes and limits of each study. Country/setting and study funding was also extracted when reported; missing or unclear items were coded as “not reported”. When multiple measures/time points were reported, we prioritised validated objective tools (e.g., GRS, SAMS, OSATS) at the earliest post-training assessment, and all others were summarised narratively. Additionally, the Oxford Centre for Evidence-Based Medicine [19] framework was applied to assess the Level of Evidence (LoE) and Level of Recommendation (LoR).

### 2.4. Quality Assessment (Risk of Bias) and Validity

Quality assessment was conducted by two reviewers (VE and TZC) who independently scored each study using the Medical Education Research Study Quality Instrument (MERSQI) [20]. The scores range from 0 to 18, across 6 domains, each with a maximum score of 3: (1) study design, (2) sampling, (3) type of data, (4) validity of evaluation instrument, (5) data analysis, and (6) outcomes. Item-level discrepancies were resolved by discussion to a consensus. The senior author independently reviewed 15% of the risk of bias assessment and found no inconsistencies.

To assess the validity of the training programmes, an established validity framework was used, incorporating content, criterion (concurrent and predictive), construct, and face validity, as described in the classical test theory literature [21].

Content validity refers to the extent to which a measurement tool accurately captures all facets of the concept it is intended to evaluate, ensuring comprehensive coverage of the theoretical domain. In the context of microsurgical training programmes, it should include aspects such as instrument and needle handling, microscope usage, the inclusion of low-, moderate- (nonbiological or biological), and high-fidelity training models, dissection, and microvascular anastomosis.

Face validity represents the degree of realism, or how closely a simulator replicates clinical situations. Construct validity reflects the simulator’s ability to differentiate between different skill levels. Criterion validity evaluates whether training performance correlates with a relevant external outcome or criterion (surgical improvement in clinical settings), either measured at the same time (concurrent) or in the future (predictive). Concurrent validity, a subtype of criterion validity, assesses the extent to which a test correlates with an established benchmark or “gold standard”; however, this form of validity could not be reliably evaluated in this review due to the absence of a universally accepted standard. Predictive validity measures how well a test can forecast future performance in clinical practice based on current training scores.

We allocated studies to syntheses based on the a priori groupings. Due to the heterogeneity of the included studies and incomplete statistics, a quantitative synthesis was not feasible. Therefore, we followed the Synthesis Without Meta-Analysis (SWiM) guidance [22] and narratively summarised the findings, using prespecified study groupings, a common synthesis metric (direction of effect: improved/no change/worse for objective performance), exploration of patterns, and robustness checks.

## 3. Results

**Figure 1:**
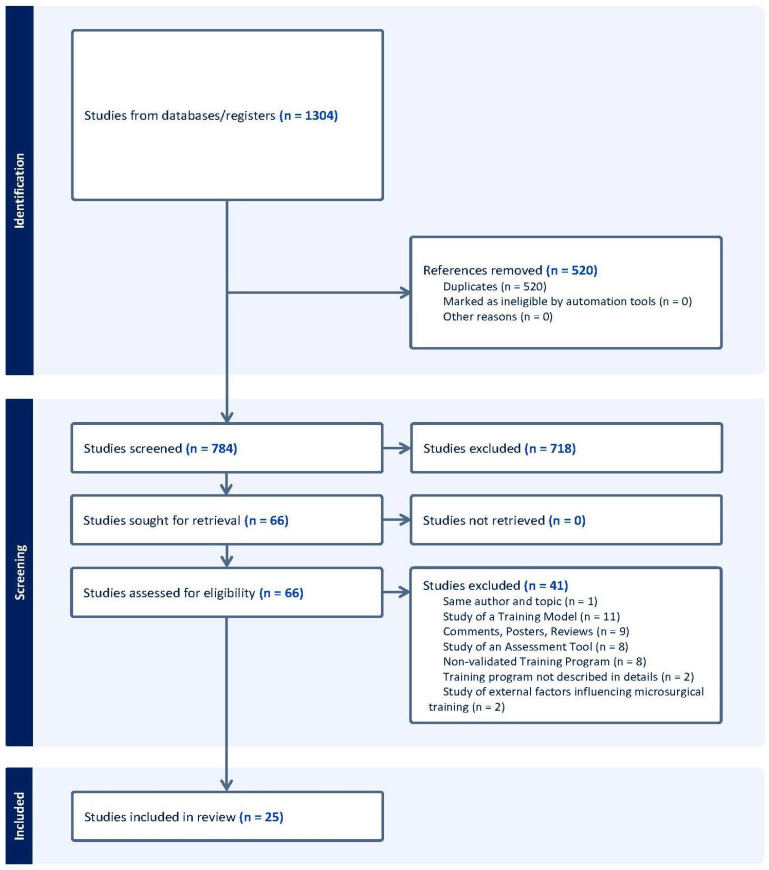
Study selection for the systematic review (PRISMA 2020 diagram). PRISMA = Preferred Reporting Items for Systematic Reviews and Meta-Analyses, where *n* = number. Records identified from databases/registers (*n* = 1304). Duplicates removed (*n* = 520; none excluded by automation). Records screened (*n* = 784) and records excluded (*n* = 718). Full texts assessed (*n* = 66) and not retrieved (*n* = 0). Full texts excluded with reasons (*n* = 41): same author/topic (1), study of a training model (11), comments/posters/reviews (9), study of an assessment tool (8), non-validated training programme (8), programme not described in detail (2), and external factors influencing microsurgical training (2). Studies included in the review (*n* = 25). Adapted from the PRISMA 2020 flow diagram.

A total of 1304 articles were identified through the search strategy, and 784 were screened after removing 520 duplicates. Following title and abstract screening, 66 articles were selected for full-text review. Of these, 41 were excluded due to them describing non-validated training programmes or because the primary focus was on validation of a training model, an assessment tool, or other external factors influencing microsurgical training, rather than evaluating a training regimen itself. Reasons for full-text exclusion were recorded and are presented in the PRISMA flow diagram (Figure 1). Comments, posters, reviews, and training programmes lacking sufficient detail were also excluded. From the 25 studies that met the inclusion criteria, data were extracted and classified into the categories presented in Table 1 and Table 2.

The reviewed articles presented a combination of basic, simulation-based and live animal training models. The training regimens varied in complexity, incorporating low-fidelity training models, such as latex strips, silicone tubes, or novel approaches like 3D-printed simulators (Geoghegan et al., 2023) [35]. These alternatives were introduced to reduce the reliance on moderate-fidelity models, such as biologic tissues, and high-fidelity models involving live animals.

### 3.1. Duration and Trainees

The training programmes were classified as either one-time courses or longitudinal curricula. The one-time courses consisted of single-day workshops (Berretti et al., 2018) [23] and workshops lasting for 5–10 consecutive days (Perez-Abadia et al., 2017; 2023) [28,29]; rapid improvement was demonstrated throughout the condensed training sessions. On the other hand, longitudinal curricula extended over several months (Chacon et al., 2020; Santyr et al., 2022; Komatsu et al., 2013; Zambrano-Jerez et al., 2024) [31,39,44,45], with training sessions scheduled weekly or biweekly. This allowed for recurrent practice aimed at progressive skill acquisition and long-term microsurgical skill retention and confidence. The one-time courses typically involved a more intensive learning approach (Lahiri et al., 2020) [10], with some studies reporting participant fatigue [28,29].

Groups sizes ranged from 5 participants (Chacon et al., 2020) [31] to 624 (Perez-Abadia et al., 2023) [29]. In 23 studies, participants included medical students, surgical residents, or senior surgeons, while Perez-Abadia et al. (2017 and 2023) [28,29] also included researchers and other nonsurgical learners. The majority of the trainees began with minimal or no prior microsurgical experience (Komatsu et al. 2013; Zambrano-Jerez et al. 2024) [39,45]. Few studies included participants with prior microsurgical skills [33], enabling comparative analysis between novices and more experienced trainees.

### 3.2. Training Programme Characteristics

The detailed training structures, procedural order, and training models used are available in Supplementary Materials S3. Face validity was the most frequently assessed type across the training programmes, followed by content and construct validity. Predictive validity was assessed in four training programmes. As no gold standard training regimen currently exists in the literature, concurrent validity was not assessed in any of the studies. Improvement in microsurgical skills was consistently reported across the studies, and was assessed with objective metrics such as the GRS, SAMS, and OSATS [39,47,48,49]. Self-directed training methods were described by Luther et al. (2019) [27], showing enhanced technical precision and reduced time to anastomosis completion. Subjective assessment tools, such as feedback surveys or self-reported confidence levels, were also described as a complementary measure alongside objective assessments. For instance, Chauhan et al. (2023) [32] reported reductions in anxiety and perceived workload, alongside objective improvements in technical performance.

### 3.3. Limitations and Evidence

The most frequently reported limitation was the small sample size of participant groups. Variability in baseline participant skill level also posed challenges in standardising outcomes and reporting the training effectiveness. Another methodological weakness was the lack of blinding in assessments and participant selection, as well as reliance on subjective assessment tools. Le Hanneur et al. (2024) [26] highlighted that the absence of advanced validation frameworks affects the value of study outcomes. Ethical and logistical challenges related to the use of live animals remain a concern. Several studies emphasised the importance of reducing reliance on this high-fidelity model in favour of low- or moderate-fidelity alternatives that can still effectively support the acquisition of microsurgical skills (Guerreschi et al., 2014; Esanu et al., 2022) [34,36]. Regarding the Levels of Evidence, most studies presented a moderate LoE (3–4), primarily due to small sample sizes or the lack of blinding. The general LoR of recommendations derived from these studies is moderate, indicating that while the programmes appear effective, further validation through larger, controlled trials is needed.

### 3.4. Quality Assessment

Across the 25 included studies, the mean MERSQI score was 12.96, with the scores ranging from 10.5 to 15.5 (SD 1.40; median 13.0 [IQR 12.0–14.0]). By design, 13/25 (52%) were single-group pre–post, 6/25 (24%) were non-randomised comparative, and 6/25 (24%) were randomised trials. Single-centre sampling predominated (23/25; 92%), with only 2/25 (8%) multi-institutional studies. The response rates were generally high (22/25; 88%). All studies used objective performance data, and data analysis was typically appropriate (25/25; 100%) and often of moderate quality (20/25; 80%). Validity evidence for the measurement instruments varied: 6/25 scored 3/3, 6/25 scored 2/3, 12/25 scored 1/3, and 1/25 scored 0/3. Outcomes were mostly at the knowledge/skills level (1.5/3–20/25; 80%), with 5/25 reporting behaviour/performance outcomes (2/3), and none reporting patient-level outcomes (3/3). We found no clear evidence of selective outcome reporting, though the incomplete reporting limited this assessment. The full table of scores, alongside the MERSQI scoring instrument [20], can be found in Supplementary Materials S4.

### 3.5. Narrative Synthesis (SWiM)

The direction of effect (classified as improved, no change, or worse) favoured the intervention in most comparisons for objective performance. Effects were more consistent in longitudinal programmes than in one-time courses, and studies with blinded assessment showed fewer discordant results. Findings were similar after excluding studies in the lowest MERSQI tertile. Ongoing heterogeneity and incomplete reporting precluded meta-analysis and formal GRADE ratings (Grading of Recommendations, Assessment, Development and Evaluations). Overall confidence in the evidence is moderated, limited by study design.

## 4. Discussions

This systematic review examined the structures, validation techniques, and effectiveness of skill acquisition in published microsurgical training programmes. The included studies presented heterogeneous training approaches, which were grouped as either one-time intensive courses or longitudinal training curricula [50,51,52] based on duration and frequency, and each with distinct advantages and limitations. Overall, we found good face and construct validities in most studies (resemble real microsurgery and distinguish skill levels), while the evaluation of criterion and predictive validity is currently lacking. Most studies were classified as low-quality regarding LoE, with small sample sizes.

It is known that organised microsurgical training provides confidence and has a positive psychological impact [53]. A clear, expected outcome across all studies, apart from the Jensen et al. (2023) Lazybox programme [37], was that microsurgical skill acquisition can be enhanced with structured training programmes that emphasise progressive learning, whether delivered as a short, intensive programme, or following a longitudinal model. Most studies demonstrated significant improvement in technical performance, frequently assessed with structured, objective tools such as the GRS, SAMS, and OSATS. Subjective measures were also reported in several studies, including confidence levels and participant feedback. Although these methods lack objective reliability, they indicated increased comfort and technical proficiency following training.

The general trend towards integrating more medium- and low-fidelity training models was also observed in this review, in line with the 3R principle of animal use in research (replacement, reduction, refinement). This training method proves to be at least as effective as the exclusive use of high-fidelity models in specific, organised, and reproducible training programmes. Guerreschi et al. (2014) [36] proposed a task trainer involving needles arranged on polystyrene when conducting training sessions for basic microsurgical instrument handling; such an approach can be performed with less, or even without, the use of live animals, while demonstrating comparable outcomes to traditional models. Despite the recent hype around emerging technologies, no validated virtual reality or augmented reality microsurgical training programmes were identified during the screening process.

With respect to validation, most studies evaluated the face, content, and construct validity, but few incorporated predictive validity, which represents the main goal of laboratory and simulation-based training. This concern, alongside the need for a gold standard microsurgical training programme that would allow for the evaluation of concurrent validity, highlights the need of more targeted future research on this topic. From the four identified studies that assess predictive validity, the strongest assessment comes from the randomised trial by Cui et al. [33], where blinded expert ratings showed higher GRS in the operative room for trainees exposed to a clinical-scenario simulator during their first patient anastomosis, albeit with only short follow-up and process-based endpoints. Santyr et al. 2022 [44] described transfer to clinical settings and skills retention, but without statistically analysing the validation and blinding the assessment. Trignano et al. 2017 [30] compared the training paths against clinical free-flap performance and satisfaction, also without blinding and with a small sample. Chauhan et al. 2023 [32] reported objective improvements using the SMaRT scale, resulting in reduced workload and anxiety, but without including direct clinical performance endpoints.

Among the reviewed training programmes, Cui et al. [33] achieved the only curriculum with level 1 (Grade A) evidence, with robust face, content, construct, and predictive validation. The next highest-validated programmes (Level 2, Grade B) also demonstrated solid validation frameworks. These included three one-time training modules [26,27,30], and six recurring training programmes [34,35,36,37,40,44]. Although these do not meet the criteria for Level 1 evidence, they feature well-defined training progressions and diverse assessment methods (e.g., SMaRT scale, SAMS, OSATS), making them strong, evidence-based references for future microsurgical training designs.

All microsurgical training programmes categorised as Level 1 and 2 share a progressive skill-based approach. They begin with basic microsurgical techniques on simple or synthetic models and progress towards more complex anastomoses on ex vivo or live animal models, minimising the use of live animals while maximising skill acquisition through alternative methods. Additionally, they emphasise repetitive practice, faculty feedback, and objective assessment. If a “gold standard” programme was to be developed, incorporating the strongest elements from these Level 1 and 2 programmes, it should include the following:

1. **Structured Progression**—Beginning with validated bench-top or synthetic models for basic microsurgical suturing, microscope accommodation, and instrument manipulation. It should progress to ex vivo models as the mainstay of training, with minimal and strategic use of live models at predefined timepoints for skill verification.
2. **Validated, Multi-faceted** Assessment—Implementation of a variety of standardised, objective measures (OSATS, GRS, SMaRT scale) to capture technical proficiency over time and across various facets of the learning curve. Ideally, the programme should also invest in confirming long-term skill retention.
3. **Longitudinal Format** (multi-session, spaced curricula, as opposed to a single time-limited course);
4. **Controlled Learning Environment and Expert Feedback**—Essential prerequisites to ensure progression happens in a controlled environment. Each participant should follow a tailored training regimen.

In an ideal scenario, in addition to the aforementioned components, a “gold standard” training programme would further benefit from the following:

5. **Integration of Criterion/Predictive Validity**—To be incorporated as ultimate, measurable objectives during clinical cases to monitor progression and provide closed-loop feedback for the tailored training regimen.
6. **An Objective Assessment Tool**—To reliably assess progression across the various stages of the programme, incorporating predefined checkpoints to determine the optimal moment for advancement. This tool should be used in conjunction with continuous expert feedback.

Items 1–4 are derived from programmes supported by higher LoE in our synthesis. By contrast, items 5–6 synthesise our findings and LoE with core principles of assessment validity, namely, the need for objective, reliable evaluation (item 5) and the need for evidence that the simulator performance predicts clinical performance (item 6), which, after all, represents the main purpose of the microsurgical laboratory training.

Translating the six-point framework into routine practice faces several barriers. Accessibility to structured microsurgical training remains a challenge in many medical centres, particularly in regions with limited funding. While one-time courses may facilitate relatively rapid skill acquisition, longitudinal training programmes appear to enhance the long-term retention of microsurgical skills. The financial burden, especially for the maintenance of high-fidelity models, remains a limiting factor. This underscores the need for current microsurgical training research and institutions to prioritise cost-effective alternatives, such as the integration of low- and medium-fidelity models within validated training programmes, in order to improve worldwide access to microsurgical training.

Regarding methodological quality, the mean MERSQI was 12.96/18 (range 10.5–15.5), indicating moderate rigour. The most common limitations were single-group pre–post designs, single-centre sampling, and the mixed instrument validity evidence, with the outcomes being concentrated at the knowledge/skills tier. (MERSQI scoring instrument, can be found in Supplementary Materials S4) [20]. Together, these factors reduce the strength of causal inference and constrain external validity; therefore, our conclusions should be interpreted as indicating consistent improvements in simulated performance rather than definitive effects on clinical outcomes. Accordingly, we prioritised objective measures, reported results for each MERSQI domain to show where studies were stronger or weaker, and used a SWiM robustness check. The overall patterns remained similar. Future trials should employ comparative designs, multi-institutional sampling, blinded assessors, and higher-level outcomes to increase the overall evidential strength and generalisability of microsurgical training.

This review is limited by English-language restriction, reliance on four databases without grey-literature/registry searches, and the inability to conduct meta-analysis due to heterogeneity. A significant challenge is the variability of the assessment tools and training methodologies present in the literature. Despite a plethora of assessment tools in the literature, while some instruments overlap, each depends on domain-specific items, such as tremor control, suture handling, or patency, and uses distinct scoring. Although validated, absolute scores and scales are not comparable; tools are therefore not interchangeable, and instrument choice likely influenced sensitivity. Programmes should justify assessment tool selection and, where feasible, report a core outcome set to enhance comparability. There is a lack of clear, objective, gold standard methodology for measuring the efficacy of microsurgical skill acquisition and retention. Firstly, a more rigorous validation framework is needed to standardise the skill assessment across different training programmes. Secondly, the majority of microsurgical research is limited by small sample sizes and a lack of long-term follow-up to assess skill retention and clinical transferability, largely due to inherent challenges in the field. Greater emphasis should be placed on incorporating predictive validity in future microsurgical skills acquisition studies, alongside the development of longitudinal studies to evaluate long-term skill acquisition and retention.

## 5. Conclusions

Standardised and validated microsurgical training programmes are essential to ensure rapid, substantial and cost-effective skill acquisition. Although noticeable improvements have been made in reducing live animal usage in training and improving access to microsurgical training, challenges remain when standardising validation methodologies and optimising cost-effective training solutions. Future research should focus on correlating skill acquisition with clinical outcomes and refining assessment methods to objectively quantify microsurgical skill acquisition. Microsurgical skill maintenance remains an unexplored area and is poised to be the next frontier for research.

## Figures and Tables

**Table 1:** One-time training courses or modules.

General	Participants			Programme Characteristics					OCEBM	
Author	No.	Type	Exp*	Duration & Frequency	Validation Type	Assessment Methods	Training Outcomes	Study Limitations	LoE	LoR
Berretti et al. (2018) [23]	30	10MS 10SR 10SS	None	3–5 h	Face Content	Round-the-clock’ as Pre-test Scoring for arterial & venous anastomoses	Patent anastomoses in 2/3rd of cases; Students & residents outperformed surgeons’ speed & scores	Small sample size Short-duration training No blinding evaluation system not validated	4	C
Bigorre et al. (2020) [24]	10	9SR 1SS	None	2 weeks 36 h total	Face Content Construct	CoTeMi grid scoring, Pre/post- assessments	Improved score & performance on end-to-end arterial and venous anastomosis	Small sample size Potential plateau effect due to learning curve saturation Subjective evaluation	4	C
Juratli et al. (2021) [25]	120	96R 20SS	90% None	2.5 days (20 h)	Face Content Construct	Pre/post self- evaluations Completion of practical tasks (e.g., microanastomoses)	84.2% performed anastomosis alone, 15% with assist, 0.8% failed. Improvement in self-reported skills	High diversity in participants’ baseline knowledge and skills Self-assessment as a subjective measure of success	4	C
Lahiri et al. (2020) [10]	58	58SR	Minimal	5 days	Face Construct content	Patency rates, Time to complete anastomoses, Utilisation rate of live rats	Improved efficiency and comparable outcomes in Group B despite reduced use of live rats	Lack of randomisation Small sample size Exclusion of left-handed participants due to ergonomic considerations.	3	B
LeHanneur et al. (2024) [26]	93	93SR	None	3 days, 6 sessions, 3 h/session	Face Content Construct	OSATS. GRS and task- specific scales TSS for knot-tying and anastomosis.	Improvements of GRS, TSS (CT group outperformed RT). Subjective: Improved confidence & comfort	Lack of blinded assessments. No time measurements Use of only one non-living model	2	B
Luther et al. (2019) [27]	25	25SR	Only clinical	5.5 ±1.4 h	Face Construct	GRS, Pre/post evaluation Anastomosis Time, blinded qualitative ratings	Patency rates improved GRS scores increased Procedure time reduced comfort level improved	Does not address live tissue properties training GRS scores may have bias Limited sample size.	2	B
Perez-Abadia et al. (2023 + 2017) [28,29]	624	595SS 29O	40.5% — None 59.5% +	5 days, 8 h/day	Face Content Construct	Evaluations during training Pre/post-assessments, OSATS Post-course surveys	Patency rates arterial—80.3%, venous— 39%, vein graft—58.1%, EtoS—82.7%, one-way-up—89.5%, continuous suture—72.6% Subjective outcomes—77.6% increased confidence and comfort	Short duration and intensity of the course lead to participant exhaustion Self-reported data may introduce bias Lack of randomised control group. Smaller sample size for advanced techniques	4	C
Trignano et al. (2017) [30]	20	20SR	None	5 days (~35–40 h total)	Face Construct Predictive	SAMS, Clinical evaluation during free flap reconstruction, and feedback surveys	Similar skill levels to live rat model group. Improved scores for dexterity, triangulation vascular anastomosis. Higher satisfaction compared to rat group.	Limited sample size Lack of blinding Dependence on fresh human placentas	2	B

No. = Number of participants; Exp* = Previous experience of participants; MS = Medical Student; SR = Surgical Resident; SS = Surgical Specialist; R = Researcher; O = Others; h = hours; OCEBM = Oxford Centre for Evidence-Based Medicine; OSATS = Objective Structured Assessment of Technical Skills; GRS = Global Rating Scale; TSS = Task-Specific Scale; SAMS = Structured Assessment of Microsurgery Skills; SMaRT = Stanford Microsurgery and Resident Training scale; UWOMSA = University of Western Ontario Microsurgical Skills Assessment; ALI = Anastomosis Lapse Index; NASA-TLX = National Aeronautics and Space Administration Task Load Index; SURG-TLX = Surgery Task Load Index; STAI-6 = Six-item State-Trait Anxiety Inventory; SOFI = Swedish Occupational Fatigue Inventory; CoTeMi = Cours de Techniques Microchirurgicales instrument; E-to-S = End-to-side anastomosis; EtoS = End-to-side anastomosis; CT = Chicken thigh; RT = regular training.

**Table 2:** Longitudinal or recurring training programmes.

General	Participants			Programme Characteristics					OCEBM	
Author	No.	Type	Exp*	Duration & Frequency	Validation Type	Assessment Methods	Training Outcomes	Study Limitations	LoE	LoR
Chacon et al. (2020) [31]	5	5SR	4+. 1 none	7 weeks, 3 h/week	Face Content Construct	SMaRT scale & ALI scoring Pre/post assessment	Improvements in SMaRT scale Error reduction (ALI) Reduced time for tasks	Very small sample size Participants’ self-reported confidence did not show substantial gains	4	C
Chauhan et al. (2023) [32]	7	7SR	none	Self-paced	Face Construct Content Predictive	SMaRT Scale NASA TaskLoad Index STAI-6	SMaRT scores improvement, reduction in anxiety. Decrease in perceived workload	Small sample size No control group Lack of long-term tracking	4	C
Cui et al. (2024) [33]	20	20SR	none	4 weeks, 40 h/week	Face Content Construct Predictive	Global Rating Scale on a 5- point Likert scale	GRS score improvement Better dexterity, visuospatial ability, and operative flow compared to the control group.	Small sample size. Possible observer and selection bias Supervision by senior surgeons may have impacted perceived outcomes.	1	A
Esanu et al. (2022) [34]	9	9MS	minimal	24 weeks, weekly	Face Content Construct	SMaRT scale Flower Petal assessment Chicken leg evaluation	Comparable skill acquisition &improvements in all participants Non-inferiority	Small group sample size Limitations SMaRT for repeated assessments Fixed 24-week training may not fit all individuals	2	B
Geoghegan et al. (2023) [35]	10	10SR	4 none, 6+	15 h (over 1 month)	Face Construct	UWOMSA tool. Pre- and post-video assessment	Quantifiable skill improvements demonstrated through UWOMSA scores.	Small sample size. Heterogeneous baseline skills Does not address dissection or flow-related skills.	2	B
Guerreschi et al. (2014) [36]	14	14SR	none	6.3 h of simulatio, 30 half-days overall	Face Content	Task completion scores Time to complete Needle integrity evaluation	48.3% reduction in live animals used. Positive participants feedback. Comparable results to traditional training	Small sample size Potential Groups selection bias, without blinding. Lack of advanced validation/assessment frameworks	2	B
Jensen et al. (2023) [37]	24	24MS	3 none, 21+	4 weeks (12 sessions, 3/week)	Face	Pre/post-tests using performance metrics; (SOFI); SURG-TLX.	No improvement in objective performance metrics Minimal reductions in fatigue and workload in subjective metrics.	No statistically significant performance improvements. Variability in baseline skills & learning curves. Potential lack of deliberate practice by participants.	2	B
Ko et al. (2015) [38]	12	12SR	none	8 weeks, weekly, 3 h/week	Face Content Construct	GRS Time to complete arterial anastomosis. Patency testing using milking	Improvement in GRS scores. Decrease in time to completion Increased patency achievement	Potential bias due to a single evaluator. Subjective assessment measures, lack of blinding Small sample size	3	C
Komatsu et al. (2013) [39]	22	22MS	none	3 months	Face Content Costruct	Hands-on assessments 1&7-day patency for live model. Positive feedback from trainees.	Passing rates: Stage1: 100%; St2: 100; St3: 86.4%; St4: 59.1%; St5: 55.0%. Positive Trainee Feedback; programme revised based on it	Use of subjective trainer evaluations. Limited generalisability to other settings. Small sample size.	3	B
Masud et al. (2017) [40]	37	37SR	none	3 months, weekly	Face Construct Content	SAMS Pre/post assessments by independent reviewers.	Improvement in SAMS Improved subjective skill levels and confidence.	Ex-vivo setting lacks the clinical decision aspect. Potential bias due to reliance on self-reported training times	2	B
Mattar et al. (2021) [41]	89	13SR 76SS	none	16 sessions, 4 h each	Face Content Construct	SAMS + dexterity, operative flow, & judgment using GRS	Objective: Progressive improvement in skills, with mean scores increasing across sessions.	No blinding in assessment Potential lack of explicit baseline skill level. Dependency on live animal models, although minimised.	3	B
Onoda et al. (2016) [42]	29	29MS	none	3 weeks, 15 days total (7–8 h/day)	Face Content Construct	Skill assessments during stages Objective measures: operating time, vascular anastomosis time	Decreased operating & vascular anastomosis time. Enhanced skill correlation between non-rat and rat stages, reducing animal use in advanced stages.	Limited sample size Lack of long-term follow- up post-training	3	C
Rodriguez et al. (2016) [43]	10	10SS	none	17 sessions~90 min each (7 months total)	Face Content Construct	OSATS Checklist Scores: specific task proficiency. Hand-Motion Analysis	Improvement in OSATS & checklist scores. Patency Rates: 100% arterial; 50% venous	Small sample size Longer operative times compared to exp. surgeons Possible variability in prior surgical training	3	B
Santyr et al. (2022) [44]	18	18SR	minimal	17 sessions (2 half-day sessions/ month)	Face Content Construct Predictive	OSATS Pre/post assessments Blind evaluation	Improvement in microsurgical skills Transferability to clinic Skills durability after 3–4 years of training	Limited sample size, significant cost of resources. Predictive validity not statistically analyzed. Lack of blinding in OR assessment	2	B
Zambrano- Jerez et al. (2024) [45]	11	11SR	none	40 h—13 sessions 3 h each	Face Content Construct	OSATS Tremor evaluation using watch & tremor analysis app	Improvements in OSATS Improved performance in specific motor skills Tremor reduction not statistically significant	Small sample size No transition to vivo models for advanced assessment Limited sensitivity of tools for tremor analysis	3	B
Zyluk et al. (2019) [46]	12	12MS	none	30 h, 15 weeks 2 h/w	Face Content	“6-stitches test”. Subjective evaluation of tightness and leakage	Improvement in suturing skills. Successful completion of 31 microsurgical anastomoses/participant.	Small sample size. Subjective and lack of advanced assessment methods	4	C

No. = Number of participants; Exp* = Previous experience of participants; MS = Medical Student; SR = Surgical Resident; SS = Surgical Specialist; R = Researcher; O = Others; h = hours; OCEBM = Oxford Centre for Evidence-Based Medicine; OSATS = Objective Structured Assessment of Technical Skills; GRS = Global Rating Scale; TSS = Task-Specific Scale; SAMS = Structured Assessment of Microsurgery Skills; SMaRT = Stanford Microsurgery and Resident Training scale; UWOMSA = University of Western Ontario Microsurgical Skills Assessment; ALI = Anastomosis Lapse Index; NASA-TLX = National Aeronautics and Space Administration Task Load Index; SURG-TLX = Surgery Task Load Index; STAI-6 = Six-item State-Trait Anxiety Inventory; SOFI = Swedish Occupational Fatigue Inventory; CoTeMi = Cours de Techniques Microchirurgicales instrument; E-to-S = End-to-side anastomosis; EtoS = End-to-side anastomosis; St = Stage.

## Data Availability

No new data were created or analyzed in this study. Data sharing is not applicable to this article.

## Supplementary Materials

The following supporting information can be downloaded at: https://www.mdpi.com/article/10.3390/jcm14217452/s1, Supplementary Materials S1: File S1—Study protocol; Supplementary Materials S2: File S2—Full search Strategies and Database summary; Supplementary Materials S3: Table S1—Data extraction table; Supplementary Materials S4: Table S2—MERSQI scoring details; and Supplementary Materials S5: PRISMA 2020—27 item checklist.

## Abbreviations

The following abbreviations are used in this manuscript:

ALI	Anastomosis Lapse Index
CoTeMi	Cours de Techniques Microchirurgicales instrument
CT	Chicken thigh
E-to-E (EtoE)	End-to-End (anastomosis)
E-to-S (EtoS)	End-to-Side (anastomosis)
GRADE	Grading of Recommendations, Assessment, Development and Evaluations
GRS	Global Rating Scale
IQR	Interquartile Range
LoE	Level of Evidence
LoR	Level of Recommendation
MRCP	Microvascular Research Center Training Program
NASA TLX	National Aeronautics and Space Administration Task Load Index
OCEBM	Oxford Centre for Evidence-Based Medicine (Levels of Evidence)
OSATS	Objective Structured Assessment of Technical Skills
PGY	Postgraduate Year (e.g., PGY-1, PGY-2, PGY-4, PGY-5, PGY-6)
PRISMA	Preferred Reporting Items for Systematic Reviews and Meta-Analyses
PTFE	Polytetrafluoroethylene
SAMS	Structured Assessment of Microsurgery Skills
SD	Standard Deviation
SIEA	Superficial Inferior Epigastric Artery
SMaRT	Stanford Microsurgical Assessment Tool
SOFI	Swedish Occupational Fatigue Inventory
STAI-6	Six-item State-Trait Anxiety Inventory
SURGTLX (SURG-TLX)	Surgery Task Load Index
SWiM	Synthesis Without Meta-analysis
TSS	Task-Specific Score
UWOMSA	University of Western Ontario Microsurgical Skills Acquisition/Assessment
