# Supplementary material for: Validated Microsurgical Training Programmes: A Systematic Review of the Current Literature
Source: J Clin Med. 2025 Oct 22;14(21):7452. doi: 10.3390/jcm14217452 (PMC12609450; doi:10.3390/jcm14217452)
Supplement: Supplementary file 1 [file jcm-14-07452-s001.zip › Supplementary File S1- Study protocol; .pdf]

Supplement S1. Protocol (PRISMA-P–aligned)

Title: Validated Microsurgical Training Programmes: A Systematic Review of Current Literature
Short title: Protocol—Validated Microsurgical Training
Registration: Not registered in PROSPERO (protocol developed a priori)
Contributors: [VE], [TZC], [AIE], [IAS], [GD], [CMFD], [TM], [DS], [VV]
Main author – [VE]; corresponding author – [VV], [GD];
Support/funding: None.
Competing interests: None declared.
Contact: Corresponding author: Victor Volovici, MD, PhD - v.volovici@erasmusmc.nl

---

1) Rationale

Validated microsurgical training programmes are widely adopted, yet methodological quality and evidence for performance improvement and transfer to clinical practice vary. A structured synthesis focusing on validation domains and objective outcomes will guide microsurgical training programmes design and research priorities.

2) Objectives (PICOS framed)

Primary objective: Determine a golden standard training programme and whether **validated microsurgical training programmes** improve **objective technical performance** among **medical students, residents, and surgeons** compared with **baseline or alternative training**.
Secondary objectives: Summarise evidence on **anastomosis patency and time metrics**, **predictive (criterion) validity** (transfer to clinical performance), **skill**, **model fidelity**, and **resources**.

---

3) Eligibility criteria (PICOS—operational)

- **Population:** Human learners (medical students, surgical trainees, practicing surgeons) undertaking microsurgical skills training.
- **Intervention: Validated microsurgical training programmes**, defined as structured training packages that report a form of objective validation of the training programme in terms of microsurgical skill acquisition.
- **Comparator:** Pre-training baseline, alternative programme/model, or none.
- **Outcomes (pre-specified):**

   - **Primary:** Objective technical performance (e.g., OSATS/SAMS/GRS or equivalent, time-normalized task metrics).
   - **Secondary:** Anastomosis patency/time; **predictive validity** (e.g., correlation with in-theatre performance or patient outcomes); skill retention; model fidelity classification (low/moderate/high); resource/cost.

- **Study designs:** RCTs, non-randomised comparative studies, and before–after single-group studies.
- **Setting/limits:** Any setting (lab, simulation centre, wet lab, clinical adjunct). **Language:** English. **Exclusions:** Reviews, editorials, case reports, descriptive commentaries; studies without a structured programme or without at least one form of objectively validating the skill retention of the training programme.

---

4) Information sources

- **Databases:** MEDLINE (Ovid), Embase (Embase.com), Web of Science Core Collection, ERIC.
- **Coverage:** Inception to **27th January 2025** (final search date).
- **Other sources:** Reference lists of included studies and relevant reviews. (No formal grey-literature or trial-registry search, due to expected low yield for education validation studies and resource constraints.)

5) Search strategy

Full strategies for each database (controlled vocabulary and keywords for microsurgery, training/skills, validation domains, and performance outcomes) are provided in **Supplement S3**. No date or study-design filters were applied beyond the language limit.

---

6) Study records

6.1 Data management

Search results were exported to a reference manager and deduplicated, then imported into **Covidence** for screening, study selection tracking, and data extraction.

6.2 Selection process

Two reviewers (**[VE]**, **[AIE]**) independently screened titles/abstracts against eligibility, followed by **independent full-text review ([VE], [TZC])**. Disagreements were resolved by **discussion** (pre-specified adjudicator: senior author **[VV]**). Reasons for full-text exclusion were recorded and are displayed in the PRISMA flow diagram.

6.3 Data collection process

Two reviewers (**[VE]**, **[TZC]**) **extracted data in duplicate** using a piloted form, as seen from the text Tables. Discrepancies were resolved by discussion; consensus values were used for analysis.

---

7) Data items (extraction fields)

- **Study descriptors:** author, year, country, setting (lab/centre), single vs multi-centre, design.
- **Participants:** learner type (students/residents/surgeons), sample size, baseline experience/skill (if reported).
- **Programme:** name/acronym, components, duration/intensity (sessions, hours), delivery (one-time vs longitudinal), supervision, feedback modality.
- **Model fidelity:** classification (low/moderate/high) and model type (non-living, ex vivo, synthetic, animal, VR/AR).
- **Assessment features:** instrument (OSATS/SAMS/GRS; version); assessor training; **assessor blinding (Y/N)**; inter-rater reliability (ICC/κ) if reported.
- **Outcomes:** primary (objective performance scores) and secondary (patency, time, predictive validity metrics, retention interval); time points (immediate, follow-up ≥3 months). **Numerical data** (means/SDs or medians/IQRs; n per group/time).
- **Analysis details:** handling of missing data, statistical tests, pre–post correlation (if given).
- **Resources/costs:** consumables, equipment, staff time (if reported).
- **Ethics/funding:** approvals, funding, conflicts.

---

8) Quality appraisal/risk of bias

8.1 Primary methodological quality tool

We used the **Medical Education Research Study Quality Instrument (MERSQI)** to appraise methodological quality (domains: study design, sampling, type of data, instrument validity, data

analysis, outcomes; total range 0–18). Two reviewers scored independently; **item-level disagreements were resolved by discussion to consensus**. We report **consensus** domain and total scores in the Supplementary Appendix.

---

9) Outcomes and effect measures

- **Primary effect measure:** For continuous objective performance scores, we planned **standardised mean differences** for pre–post within-groups and between-group contrasts where applicable.
- **Secondary measures:** Mean differences for time/patency metrics; correlations or comparative measures for **predictive validity**; proportions for patency.

---

10) Narrative synthesis

Since the heterogeneity of the data and meta-analysis was not feasible, we followed **SWiM** guidance:

1. **Pre-specified study groupings:** (a) programme type: **one-time** vs **longitudinal**; (b) **model fidelity** (low/moderate/high); (c) **assessor blinding** (yes/no).
2. **Common synthesis metric: direction-of-effect** (improved/no change/worse) for the primary outcome.
3. **Heterogeneity exploration:** compare patterns across groupings and by instrument type (objective vs subjective).
4. **Robustness checks:** remove low-quality studies (if there are)

---

11) Subgroups

- **Subgroups:**
  - Programme (one-time vs longitudinal).
  - **Model fidelity** tier.
  - **Assessor blinding** (Y/N).
  - Learner experience (novice vs intermediate/advanced).

---

12) Certainty (confidence in evidence)

We will rate certainty for key outcomes and present **Summary-of-Findings** tables (primary outcome; predictive validity). If GRADE cannot be completed, we will report this as a deviation with rationale and provide a qualitative statement of confidence using MERSQI and consistency.

---

13) Amendments & deviations

This protocol was developed a priori but **not registered**. Any changes made after protocol finalisation are documented here and in **Supplement** with date, reason, and potential impact on findings.

---

14) Patient/public involvement

None (education intervention review).

---

15) Ethics and dissemination

No ethics approval required (secondary analysis of published data). Results will be disseminated via a peer-reviewed journal and conference presentations. **Data and materials** will be shared in **Supplement**.

---

16) Roles (CRediT taxonomy)

- **Conceptualization/Methodology:** [VE], [VV], [GD], [CMFD]
- **Search strategy:** [VE], [VV], with librarian input
- **Screening/Selection:** [VE], [AIE]
- **Data extraction:** [VE], [TZC]
- **Quality appraisal (MERSQI):** [VE], [TZC] (consensus)
- **Synthesis:** [VE], [VV], [IAS]
- **Writing – original draft:** [VE], [VV], [GD], [TZC], [IAS]
- **Writing – review & editing:** [VE], [TZC], [AIE], [IAS], [GD], [CMFD], [TM], [DS], [VV]
- **Supervision:** [VV]

17) Appendices referenced

- **Supplement S1:** Study Protocol
- **Supplement S2:** Full search strategies (database-specific)
- **Supplement S3:** Detailed Validated Microsurgical Training Programmes:
- **Supplement S4:** MERSQI domain & total scores per study; bias-domain mapping figure
