# Supplementary material for: Validated Microsurgical Training Programmes: A Systematic Review of the Current Literature
Source: J Clin Med. 2025 Oct 22;14(21):7452. doi: 10.3390/jcm14217452 (PMC12609450; doi:10.3390/jcm14217452)
Supplement: Supplementary file 1 [file jcm-14-07452-s001.zip › Supplementary File S2- Full search strategies & database summary.pdf]

Annex 2. Databased Search string used and Database Search Summary

medline 599

(Microsurgery / OR (microsurg* OR microneurosurg* OR micro-neurosurg* OR supermicrosurg* OR (micro* ADJ3 surg*) OR microanastomo* OR micro*-anastomo* OR ((microvascul* OR micro-vascul*) ADJ3 surg*)).ab,ti,kf.) AND (Learning/ OR Simulation Training/ OR (training* OR ((surg* OR simulat*) ADJ3 (training OR education* OR skill* OR learning*))).ab,ti,kf. OR (training OR education* OR skill* OR learning*).ti.) AND (Validation Study / OR Validation Studies as Topic / OR Program Evaluation / OR Reproducibility of Results / OR (validat* OR validit* OR effectiv* OR ((program* OR model OR development* OR framework*) ADJ3 evaluat*)).ab,ti,kf.) AND english.la.

embase 617

(microsurgery/exp OR (microsurg* OR microneurosurg* OR micro-neurosurg* OR supermicrosurg* OR (micro* NEAR/3 surg*) OR microanastomo* OR micro*-anastomo* OR ((microvascul* OR micro-vascul*) NEAR/3 surg*)):Ab,ti,kw) AND ('surgical training'/de OR training/de OR learning/exp OR 'skills training'/de OR 'simulation training'/de OR (training* OR ((surg* OR simulat*) NEAR/3 (training OR education* OR skill* OR learning*))):ab,ti,kw OR (training OR education* OR skill* OR learning*):ti) AND ('validation study'/de OR 'validation process'/de OR 'program effectiveness'/de OR 'program evaluation'/de OR validity/exp OR (validat* OR validit* OR effectiv* OR ((program* OR model OR development* OR framework*) NEAR/3 evaluat*)):ab,ti,kw) NOT ([conference abstract]/lim AND [2000-2022]/py) AND [english]/lim

web of science 77

TS=((microsurg* OR microneurosurg* OR micro-neurosurg* OR supermicrosurg* OR (micro* NEAR/2 surg*) OR microanastomo* OR micro*-anastomo* OR ((microvascul* OR micro-vascul*) NEAR/2 surg*))) AND (TS=(training* OR ((surg* OR simulat*) NEAR/2 (training OR education* OR skill* OR learning*))) OR TI=(training OR education* OR skill* OR learning*)) AND TI=((validat* OR validit* OR effectiv* OR ((program* OR model OR development* OR framework*) NEAR/2 evaluat*))) AND DT=(article) AND LA=(english)

ERIC 11

((microsurg* OR microneurosurg* OR micro-neurosurg* OR supermicrosurg* OR (micro* ADJ3 surg*) OR microanastomo* OR micro*-anastomo* OR ((microvascul* OR micro-vascul*) ADJ3 surg*)).ab,ti.)

**Database searched**	**Platform**	**Years of coverage**	**Records**	**Records after duplicates removed**
Medline ALL	Ovid	1946 - Present	599	598
Embase	Embase.com	1971 - Present	617	171
Web of Science Core Collection*	Web of Knowledge	1975 - Present	77	5
ERIC	Ovid	1965 - Present	11	10
**Total**			**1304**	**784**

*Science Citation Index Expanded (1975-present) ; Social Sciences Citation Index (1975-present) ; Arts & Humanities Citation Index (1975-present) ; Conference Proceedings Citation Index- Science (1990-present) ; Conference Proceedings Citation Index- Social Science & Humanities (1990-present) ; Emerging Sources Citation Index (2005-present). No other database limits were used than those specified in the search strategies
