# Supplementary material for: Validated Microsurgical Training Programmes: A Systematic Review of the Current Literature
Source: J Clin Med. 2025 Oct 22;14(21):7452. doi: 10.3390/jcm14217452 (PMC12609450; doi:10.3390/jcm14217452)
Supplement: Supplementary file 1 [file jcm-14-07452-s001.zip › Supplementary Table S1- Data extraction table; .pdf]

Annex 3. Detailed Validated Microsurgical Training Programmes:

No.	Title	Described	Training Programme detailed description
1	Pilot study on microvascular anastomosis: performance and future educational prospects	Berretti et al. (2018)	**1. Pre-Course Preparation**<br>**Brief theoretical introduction** (Proper posture, Microscope usage, handling of micro instruments, basic suture principles)<br>**2. Training Phases**<br>**Phase 1:** Pre-Test ('Round-the-Clock' Exercise)<br>**Phase 2:** Simulation Training with macroscopic models: Silicone tubes as surrogate arteries and finger gloves as surrogate veins,<br>**Phase 3:** Microscopic Training: rooster vessels (2.5-3.5 mm) simulating human microvascular anatomy<br>**Phase 4:** Microanastomosis Practice: end-to-end arterial and venous anastomosis. Time limit of 60 minutes/anastomosis.
2	Fast-track teaching in microsurgery	Bigorre et al. (2020)	**Week 1**<br>**Day 1:** Suture on inert material and Shirataki noodles<br>**Day 2:** Rat dissection (Aorta and vena cava, Carotid artery, Renal pedicle, Uterus, Sciatic Nerve)<br>**Day 3:** Rat uterus suture, Transverse aorta suture<br>**Day 4:** Transverse aorta suture, Flanged Sciatic Nerve Suture<br>**Day 5:** Carotid artery suture x 2<br>**Week 2**<br>**Day 6:** Assessment session: Carotid artery + flanged carotid artery<br>**Day 7:** Kidney graft (renal artery and vein) + Transverse carotid suture<br>**Day 8:** Jugular vein graft on subrenal aorta + Carotid artery suture<br>**Day 9:** Renal artery and carotid artery sutures + Optional: Omega bridge (jugular vein) on subrenal aorta.<br>**Day 10:** Final examination: Transverse suture abdominal aorta and carotid artery + Optional: Renal artery and vein sutures<br>**Day 11:** Remedial session (if needed for students who failed the final exam).
4	Fundamentals of Microsurgery: A Novel Simulation Curriculum Based on Validated Laparoscopic Education Approaches	Chauhan et al. (2023)	**Task 1:** Rubber Band Transfer: Sequentially transfer 25 rubber bands between the right and left hand within 2 minutes.<br>**Task 2:** Coupler Tine Grasping: Grasp venous coupler tines in a predetermined order using angled forceps within 1 minute.<br>**Task 3:** Glove Laceration Repair a 1-cm laceration in a latex glove stretched within 3 minutes.<br>**Task 4:** Synthetic Vessel Anastomosis on synthetic vessels within 12 minutes<br>**Task 5:** Synthetic Vessel Anastomosis in a 5-cm deep cavity using synthetic vessels within 12 minutes.

3	Pretest and Posttest Evaluation of a Longitudinal, Residency-Integrated Microsurgery Course	Chacon et al. (2020)	**Weeks 1–5: Synthetic models for skill-building (e.g., rubber glove suturing, end-to-end anastomosis on synthetic tissue).**<br>**Week 1 (Introduction to Microsurgery)**: Precourse: Acland's Basic Microsurgery Video + Theory + 1.1: Peeling a grape + 1.2: Suturing a rubber glove<br>**Week 2 (Flap Classification and End-to-End Anastomosis):** Theory + 2.1: End-to-end anastomosis<br>**Week 3 (Flap Failure and Minimising Risk):** Theory + 3.1: End-to-end anastomosis using corner stitches + 3.2: End-to-end anastomosis, back wall first<br>**Week 4 (Preoperative Planning and End-to-Side Anastomosis):** Theory + 4.1: End-to-side anastomosis<br>**Week 5 (Breast Reconstruction and Timed Sessions):** Theory + 5.1: EtoE using double clamps + 5.2: EtoE using single clamp, corner stitches + 5.2: EtoE using single clamp, back wall first + 5.4: EtoE (timed session)<br>**Weeks 6–7: Live rodent models for advanced procedures (e.g., carotid artery anastomosis, femoral artery dissection).**<br>**Week 6 (Flaps for Extremity Reconstruction and In Vivo Models):** Theory + 6.1: Carotid dissection + 6.2: Left jugular vein end-to-end with coupler device + 6.3: Left common carotid artery EtoE anastomosis with double clamps + 6.4: Left inguinal dissection + 6.5: Left femoral artery EtoE anastomosis with single clamps + 6.6: Right carotid dissection + 6.7: Right neck EtoS (external jugular to common carotid)<br>**Week 7: Final Assessments and Certification**: Review of Theory + Examinations: 7.1 = Multiple-choice examination, 7.2 = Oral examination, 7.3 = Practical examination (Left common carotid artery dissection, Left common carotid artery EtoE, Raising of right groin flap, EtoE anastomosis of groin flap onto common carotid artery, Neurorrhaphy of right sciatic nerve)Weekly reading assignments for theoretical preparation.
6	Reduction of the number of live animals used for microsurgical skill acquisition: an experimental randomized non-inferiority trial	Esanu et al. (2022)	**Week 1:** Flower petal suturing, end-to-end chicken leg femoral artery anastomosis<br>**Week 2:** Flower petal suturing, 2x end-to-end chicken leg femoral artery anastomoses<br>**Week 3:** Flower petal suturing<br>**Week 4:** End-to-end chicken leg femoral artery anastomosis, end-to-side chicken leg femoral artery anastomosis<br>**Week 5:** Flower petal suturing, end-to-side chicken leg femoral artery anastomosis<br>**Week 6:** End-to-End Chicken Leg Femoral Artery Anastomosis<br>**Week 7:** End-to-End Chicken Leg Femoral Artery Anastomosis, Flower Petal Suturing<br>**Week 8:** End-to-Side Chicken Leg Femoral Artery Anastomosis<br>**Week 9:** End-to-End Chicken Leg Femoral Artery Anastomosis, End-to-End Chicken Leg Femoral Vein Anastomosis<br>**Week 10:** End-to-End Chicken Leg Femoral Artery Anastomosis, End-to-Side Chicken Leg Femoral Artery Anastomosis, Flower Petal Suturing<br>The training programme was repeated respecting this order until 24 weeks. The weekly sessions were supplemented by training on live rats every 4, 6, or 8 weeks depending on the group.
5	Innovative Clinical Scenario Simulator for Step-by-Step Microsurgical Training	Cui et al. (2024)	**Week 1:** Basic Training - Dissection, Adventitial stripping, Knot-tying techniques, Placement of interrupted sutures, patency testing, bleeding control .<br>**Week 2:** Training with Inner Baffling Rods (Simulate narrow and deep surgical spaces).<br>**Week 3:** Training with Exterior Baffling Rods - Train hand steadiness and instrument handling without support.<br>**Week 4:** Training with Pulsating Platform (Mimics respiratory movements with a pulsation rate of 20 beats per minute and a vertical excursion of 5 mm). Focus: Performing vascular anastomoses under simulated respiratory motion.<br>**Final Assessment:** After completing the four-week training:<br>• Trainees performed microvascular anastomoses on real patients under supervision.<br>Performance was evaluated using the Global Rating Scale (GRS)

7	Utilization of a 3D Printed Simulation Training Model to Improve Microsurgical Training	Geoghegan et al.(2023)	Training Platform: Konjac noodle + 3D-printed platform with Polylactic Acid, incorporating "Round-the-clock" exercise, Rib simulator, handling the needle, and 3D-printed clamps. The curriculum is progressive, with four levels of increasing technical difficulty:<br><br>**Level 1:** Nerve Coaptation - Basic suturing skills with simulated nerve repair.<br>**Level 2:** Front Wall Simple Anastomosis - Practice on the front wall of the vessel only.<br>**Level 3:** Back Wall Simple Anastomosis – Suturing only the back wall of the vessel for better depth perception.<br>**Level 4:** Full Anastomosis at Depth - Suturing both front and back walls with restricted access using the rib model
8	Reducing the number of animals used for microsurgery training programs by using a task-trainer simulator	Guerreschi et al.(2014)	**Simulator Device** (task trainer made of sewing needles arranged in circular patterns on a polystyrene base)<br>**Simulation Programme Exercises**<br>**Exercise 1:** Maneuver a threaded needle forehand through two needles set 1 mm apart and tie five knots in different directions.<br>**Exercise 2:** Perform the same manipulation as Exercise 1, but backhand.<br>**Exercise 3:** Alternate forehand and backhand maneuvers through the eyes of eight sewing needles vertically oriented on a polystyrene base.<br>**Exercise 4:** Maneuver the needle forehand or backhand through a consecutive sequence of needles positioned in a circular arrangement.<br>**Exercise 5:** Maneuver the needle in a regular pattern through the eyes of 24 needles arranged in a double-circle configuration. Students spent an average of 6.3 hours to complete all tasks. Trainees must accumulate 15 stars to move to live tissue training. Stars are awarded based on task performance (time, precision, and needle condition).<br>**Live Animal Training:** Perform vascular anastomoses (carotid artery, jugular vein, aorta, vena cava, carotid and internal jugular shunts, and carotid bypass).
9	The LazyBox Educational Intervention Trial: Can Longitudinal Practice on a Low-Fidelity Microsurgery Simulator Improve Microsurgical Skills?	Jensen et al. (2023)	**LazyBox Microsurgical Trainer (Only the treatment group trained)**<br>**Maze Task:** Using a 5-0 silk suture, participants were required to pass the suture continuously through 12 adjacent sewing needles placed in sequence three times.<br>**Plastic Straw Anastomosis Task:** Participants performed an anastomosis by tying four circumferential knots around a plastic straw.<br>**Evaluation:** Before and after the training programme, all participants (control and treatment groups) were assessed on: Tying sutures on cut vessel loops, Suturing on red rubber tubing and Suturing on synthetic blood vessels.

10	Microsurgical training course for clinicians and scientists: a 10-year experience at the Münster University Hospital	Juratli et al. (2021)	**Step 1:** Latex Diaphragm: single-knot sutures are placed with polypropylene 5-0, spaced at 5 mm intervals on Latex glove.<br>**Step 2:** Pig Skin is attached to a styrofoam board, incisions (2x4 cm) are made, and continuous sutures at 5 mm interstitch intervals.<br>**Step 3:** Glove Finger Anastomosis: A latex glove finger is transected and sutured with continuous stitches, ensuring proper posterior and anterior wall alignment.<br>**Step 4:** Anastomosis of Pig Aorta: pig aorta is transected, and a continuous anastomosis is performed using polypropylene 6-0.<br>**Step 5:** Coordination Model Under the Microscope: Participants practice stitching on a glove-based card model under a surgical microscope with varying magnifications<br>**Step 6:** Dummy Model – Part I: A plaster roll is covered with a surgical glove. A figure is drawn on the glove, incised, and stitched with single-knot sutures<br>**Step 7:** Dummy Model – Part II: The latex tube created in Step 6 is transected, corner sutures are placed, and posterior and anterior walls are sewn<br>**Step 8:** Cold-Stored Small Vessels: Frozen rat aorta segments are thawed and used to practice continuous vascular anastomoses.<br>**Step 9:** Anastomosis of Pig Coronary Artery: Pig coronary arteries are sutured end-to-end.<br>**Step 10:** Live Rat Model: Participants perform end-to-end anastomoses on the infrarenal aorta and vena cava of anesthetized rats.
11	Effectiveness of a Microvascular Surgery Training Curriculum for Orthopaedic Surgery Residents	Ko et al. (2015)	**Week 1: Introduction and Basics.** Suturing on a latex glove model to simulate vessels.<br>**Week 2: Transition to Live Model.** Introduction to live animal model (rat femoral artery).<br>**Weeks 3-7: Repeated Practice.** Arterial or venous EtoE anastomosis on the live rat model during each session.<br>**Week 8: Final Evaluation.** Final attempt at end-to-end arterial anastomosis on the live rat femoral artery.
12	Evaluation of the Microvascular Research Center Training Program for Assessing Microsurgical Skills in Trainee Surgeons	Komatsu et al. (2013)	**Stage 1: Basic Microsurgical Skills.** <u>Task:</u> Anastomose silicone tubes (1 mm in diameter). 8 sutures needed (revised programme 2008). Time limit for anastomosis: less than 20 minutes<br>**Stage 2: Practical Training on Chicken Blood Vessels.** <u>Task:</u> Anastomose blood vessels from chicken carcasses. 8 sutures. Time limit for anastomosis: less than 20 minutes<br>**Stage 3: Live Rat Femoral Vessels.** <u>Task:</u> Perform anastomosis of the femoral artery and vein of live rats. Achieve a 1-day patency rate of >80%.<br>**Stage 4: Replantation of Rat Free Flaps.** <u>Task:</u> Replant superficial inferior epigastric artery (SIEA) flaps in rats. Achieve a 7-day success rate of >80% (updated in 2008 from 60%).<br>**Stage 5: Advanced Microsurgical Techniques.** <u>Task</u>: Successfully perform one advanced tissue transplantation and replantation from: Face Allotransplantation (External carotid artery and jugular vein anastomosis); Penis Isotransplantation (Anastomosis of dorsal penile vein and corpus spongiosum to the femoral vessels). Other Cases: Lymphatic vessel grafts, forelimb replantation, testis transplantation, and more.

13	Maintaining Effective Microsurgery Training with Reduced Utilization of Live Rats	Lahiri et al. (2020)	Day 1: Familiarisation with microsurgical instruments and basic suturing techniques.<br><u>Training Models:</u> High-fidelity latex strip model (DS Microtrainer system) + 2mm synthetic tubes.<u>Activities:</u> Video demonstrations of microsurgical techniques. Hands-on practice of basic suturing on synthetic models.<br>Day 2: Transition to anastomosis practice on appropriate models. Group A:<br>Training directly on live rat models (femoral vessel anastomosis).<br>Group B: Continued practice on synthetic tubes (Lifelike Biotissue). Introduced to ex-vivo chicken thigh model for femoral vessel anastomosis.<br>Day 3: Introduction to live rat models for both groups.<br><u>Training Models:</u> High-fidelity latex strip model. Live rat models for femoral artery and vein anastomosis. <u>Activities:</u> Continued practice of anastomosis techniques. Transition to live rat models for hands-on training.<br>Day 4: Consolidation of anastomosis skills with live rat practice.<br>Group A: Full-day practice on live rat femoral vessels.<br>Group B: Split training session: Practice on high-fidelity latex strip models & Practice on live rat femoral vessels.<br>Day 5: Final practice and assessment.<br><u>Training Models:</u> High-fidelity latex strip model. Live rat femoral vessels (right and left arteries and veins). <u>Activities:</u> test setting for participants on live rat femoral vessels.
14	Nonliving versus Living Animal Models for Microvascular Surgery Training: A Randomized Comparative Study	LeHanneur et al.(2024)	**Session 1 (Theoretical + Hands-On Training):** Theoretical lessons on microsurgery principles and tools. Knot-tying practice using woven gauze. Microvascular anastomoses practice on silicone tubes.<br>**Session 2 (Hands-On Training):** Advanced practice in performing microvascular anastomoses on silicone tubes oriented in different ways.<br>**Session 3 (Hands-On Training - First Animal Model):**<br><u>RT Group:</u> Performed end-to-end anastomoses of the carotid artery and external jugular vein on a living rat model.<br><u>CT Group:</u> Performed end-to-end anastomoses of the femoral artery and vein on a non-living chicken thigh model.<br>**Session 4 (Evaluation - Living Rat Model):** Trainees performed end-to-end anastomosis of the subrenal abdominal aorta on a living rat model.<br>**Session 5 (Hands-On Training - Living Rat Model):** Performed end-to-end anastomoses of the femoral artery and vein on a living rat model.<br>**Session 6 (Evaluation - Living Rat Model):** Trainees performed bypass of the subrenal abdominal aorta using the right external jugular vein as a venous graft on a living rat

15	Achieving Microsurgical Competency in Orthopaedic Residents Utilizing a Self-Directed Microvascular Training Curriculum	Luther et al. (2019)	**Module 1: Basic Microsurgery Setup and Instrument Handling** Task: Practice passing suture and needle between instruments under magnification.<br>**Module 2: Knot Tying and Suture Placement.** Task: Practice of knot tying and suture placement on latex glove models mounted on a cardboard frame. Proper apposition of tissue edges. Also Using the nondominant hand effectively. Progression from 6-0 nylon suture to finer 9-0 nylon suture for increased precision.<br>**Module 3: Anastomosis on Teflon Tubing** Utilisation of 2-mm inner diameter Teflon (PTFE) tubing to simulate vascular repair.Tasks: Placement of tissue approximators. Suturing the front and back walls of the tube. Practice interrupted and continuous suture configurations. Conducting seal checks on completed anastomoses.<br>**Module 4: Final Anastomosis on Synthetic Microvessels:** Completion of microvascular anastomosis on a 1-mm synthetic vessel designed to replicate digital artery characteristics (appearance, texture, and resistance).
16	Microsurgery simulation training system and set up: An essential system to complement every training programme	Masud et al. (2017)	**Step 1: Needle Dexterity (Weeks 1-3)**<br>"Round the clock" task: Passing 8-0 needle through sewing needle eyes + "In out up down" task: Weaving suture through Mepitel dressing. The tasks are timed in order to progress to the next step.<br>**Step 2: Economy of Movement (Weeks 4-6)**<br>"All tied up" task: Tying knots with restricted hand movement + "Suturing at angles" task: Suturing at various angles with quality assessment. The tasks are timed in order to progress to the next step.<br>**Step 3: Operative Flow (Weeks 7-9)**<br>"Stars the limit" task: Chicken femoral artery dissection and anastomosis, Acland test. Fine tissue dissection on grape skin. The tasks are timed and scored in order to progress to the next step.<br>**Step 4: Operative Judgement (Weeks 10-12)**<br>Vessel discrepancy anastomosis. End-to-side anastomosis. Arteriotomy Freestyle – Wk11. Final SAMS assessment
17	Structured evaluation of a comprehensive microsurgical training program	Mattar et al. (2021)	**1. Rubber Glove Suturing (2 Sessions):** Participants practiced progressively thinner suture lines, starting with 7-0 sutures and advancing to 10-0 sutures.<br>**2. Chicken Thigh Training (2 Sessions):** Performed arterial, venous, and nerve end-to-end anastomoses on chicken thighs.<br>**3. End-to-End Anastomosis on Live Rat Femoral Arteries (5 Sessions):** Participants performed arterial end-to-end anastomoses on live rat femoral arteries.<br>**4. End-to-End Anastomosis on Live Rat Femoral Veins (5 Sessions):** Participants performed venous end-to-end anastomoses on live rat femoral veins.<br>**5. Arterial Graft Interposition in Live Rat Femoral Arteries (1 Session):** Participants performed arterial graft interposition, where a segment of an artery is replaced with a graft.<br>**6. End-to-Side Anastomosis from Live Rat Femoral Artery to Vein (1 Session):** Participants performed arterial-to-venous end-to-side anastomoses.<br>**Total: 16 sessions,** structured progressively from basic to advanced tasks.

18	Analysis of 10-Year Training Results of Medical Students Using the Microvascular Research Center Training Program	Onoda et al. (2016)	**Day 1: Stage 1:** Perform anastomosis on 1-mm silicone tubes (25 minutes per trial, 5 trials = 150 minutes).<br>**Day 2: Stage 1:** Continue anastomosis on silicone tubes (25 minutes per trial, 10 trials = 150 minutes).<br>**Days 3–4: Stage 2:** Perform anastomosis on 1–2 mm blood vessels in chicken wings (50 minutes per trial, 5 trials = 250 minutes).<br>**Days 5–6: Stage 3:** Perform supermicrosurgery on <1 mm blood vessels in chicken wings (50 minutes per trial, 5 trials = 250 minutes). A success rate of more than 80% in 5 consecutive SIEA flap survival is required to complete the program<br>**Days 7–10: Stage 4:** Perform vascular anastomosis on femoral arteries and veins in rats (120 minutes per trial, 2 trials per day = 240 minutes).<br>**Days 11–15: Stage 5:** Perform free superficial epigastric artery (SIEA) flaps in rats (120 minutes per trial, 2 trials per day = 240 minutes). Optional: Advanced stages offered to trainees who complete the MRCP, including facial transplantation, limb transplantation, or lymphaticovenular anastomosis in rats
19	Frankfurt microsurgery course: the first 175 trainees	Perez-Abadia et al.(2017)	**Day 1: Foundational Skills:** Basic posture and ergonomics. Handling and care of microsurgical instruments. Knot-tying exercises on non-living models (e.g., synthetic or plastic). Introduction to microscope operation and basic suture techniques.<br>**Day 2:Arterial Anastomosis on Live Models:** Practice performing end-to-end arterial anastomoses using femoral arteries of live rats. Emphasis on vessel preparation, suturing technique, and achieving patency.<br>**Day 3: Venous Anastomosis on Live Models:** Performing end-to-end venous anastomoses on femoral veins. Addressing challenges unique to venous anastomoses, such as vessel fragility and thrombosis prevention.<br>**Day 4: Vein Graft Anastomosis:** Interpositional vein graft technique using live rat models. Key learning objectives: precision, alignment, and maintaining vessel patency.<br>**Day 5: Advanced Techniques** End-to-side arterial and venous anastomoses on live models.<br>Optional tasks for advanced trainees: One-Way-Up Arterial Anastomosis: Suture from back to front without flipping the clamp. Continuous Suturing Technique: Performing continuous sutures on 1 mm vessels, maintaining lumen diameter.
20	Intensive hands-on microsurgery course provides a solid foundation for performing clinical microvascular surgery	Perez-Abadia et al.(2023)	**Day 1:** Basic posture, instrument handling, and glove-rubber suture exercises.<br>**Day 2:** End-to-end femoral arteriy anastomosis on live rats.<br>**Day 3:** End-to-end femoral vein anastomosis on live rats.<br>**Day 4:** Interpositional Vein graft technique on live rats.<br>**Day 5:** End-to-side anastomosis on live rats.<br>Trainees progress to more advanced techniques, with additional tasks like one-way-up and continuous suturing based on individual proficiency.

21	Microsurgery Workout: A Novel Simulation Training Curriculum Based on Nonliving Models	Rodriguez et al.(2016)	**Sessions 1-4: Suturing on Synthetic Models:** Perform five series of three stitches using a latex glove model in the first two sessions.<br>**Sessions 5-6: Vessel Dissection and Preparation (Chicken Wing Model)**: Vessel identification, ligation, coagulation of branches using bipolar forceps, setting up approximation clamps, vessel transection, dilation, and adventicectomy. Perform twice on brachial arteries and twice on brachial veins in each session.<br>**Sessions 7-8: Arterial Anterior Wall Suturing (Chicken Thigh Model):** Two stay sutures placed at 180 degrees, followed by interrupted stitches. Perform on the anterior wall of the femoral artery three times during each session.<br>**Sessions 9-10: Perform complete arterial anastomosis on the femoral artery using interrupted stitches.**<br>**Sessions 11-13: Perform complete arterial anastomosis on the brachial artery:** Closure of the posterior wall was performed after turning the clamp. Each procedure was repeated twice per session.<br>**Sessions 14-16: Complete Venous End-to-End Anastomosis:** Perform complete venous anastomosis on the brachial vein twice per session.<br>**Session 17: Combined Arterial and Venous End-to-End Anastomosis:** Perform both arterial and venous end-to-end anastomosis on the chicken wing model as a final assessment before moving to the live rat model.<br>**Each session was standardised to 90 minutes.**
22	High-fidelity, simulation-based microsurgical training for neurosurgical residents	Santyr et al. (2022)	**Module 1: Introduction to Microsurgery**<br>Perform a 6-0 monofilament running suture along a 3-cm incision (3 repetitions). Complete 20 interrupted 10-0 monofilament sutures in a simulated skin model.<br>**Module 2: Intermediate Microvascular Dissection**<br>Dissect the brachial artery in a perfused cadaveric duck wing model. Observe instructional videos on femoral vessel exposure in live rats.<br>**Modules 3-5: Live Rat Model for Microvascular Anastomosis -** Each of these modules is repeated five times.<br>**Module 3: Artery-to-Artery Anastomosis:** Perform end-to-end anastomosis on the femoral artery.<br>**Module 4: Vein-to-Vein Anastomosis:** Perform end-to-end anastomosis on the femoral vein.<br>**Module 5: Artery-to-Vein Anastomosis:** Perform end-to-side anastomosis between the femoral artery and vein.<br>Total = 17 sessions
23	Microsurgical Training with the Three-Step Approach	Trignano et al. (2017)	**Step 1. Basic Microsurgical Skills:**<br>On latex gloves, multiple incisions are made in the latex at different angles (30°, 180°, and 270°) to practice sutures.<br>**Step 2: Intermediate Microsurgical Skills:**<br>On Endovascular prostheses. Trainees begin with larger prostheses and gradually move to smaller ones.Techniques practiced include: Triangulation Technique, Posterior Wall Technique, continuous and interrupted suturing techniques.<br>**Step 3: Advanced Microsurgical Skills**:<br>On fresh human placentas obtained from the delivery room. Procedures include Vessel isolation and dissection, Adventectomy (removal of the adventitia layer), End-to-end vascular anastomosis. If the placenta is used within 3–4 hours post-delivery, trainees can observe vessel reperfusion after successful anastomosis.

24	Novel and easy curriculum with simulated models for microsurgery for plastic surgery residents: reducing animal use	Zambrano-Jerez et al. (2024)	**1. Initial Assessment (Diagnostic Session):** Participants performed baseline exercises on non-living and ex-vivo models.<br>**2. Sessions 1–2: Basic Skills Training:** Basic exercises: Cutting, suturing, and knot-tying on latex and rose petals. Practice on non-living models: Manipulation of cigarette components and creating gauze figures using sutures.<br>**Sessions 3–4: Placental Model Training:** Vascular dissection and termino-terminal arterial anastomosis on dye-perfused placental models.<br>**Sessions 5–6: Chicken Thigh Model Training:** Vascular dissection and termino-terminal arterial anastomosis. Practice on realistic blood vessel diameters (1–2 mm arteries and 2–3 mm veins).<br>**Session 7: Advanced Venous Anastomosis:**Termino-terminal and termino-lateral venous anastomoses using the placenta model.<br>**Sessions 8–9: Chicken Thigh – End-to-Side Anastomosis:** Dissection and termino-lateral arterial anastomosis.<br>**Sessions 10–11: Chicken Thigh – Advanced Venous Anastomosis:** Termino-terminal venous anastomosis.<br>**Sessions 12–13: Chicken Thigh – End-to-Side Venous Anastomosis** Final exercises on termino-lateral venous anastomoses.<br>**Final Assessment:** Participants performed the same exercises from the diagnostic session.<br>**Each session starts with a theoretical module and an explanatory video.**
25	Undergraduate microsurgical training: a preliminary experience	Zyluk et al. (2019)	**Start of basic training**<br>**Week 1 (2 hours):**Introduction to the programme. Familiarisation with the microscope and instruments. Suturing on a rubber glove.<br>**Week 2 (4 hours):** Suturing on a rubber glove. Preparation of structures on a chicken thigh. Introduction to nerve suturing techniques. Chicken femoral nerve suture (1 anastomosis per person).<br>**Week 3 (6 hours):** Suturing on a rubber glove (as warm-up). Continued nerve suturing. Introduction to microvascular suturing techniques. 2 anastomoses per person.<br>**Week 4 (8 hours):** Chicken femoral nerve suturing. Chicken femoral artery anastomosis (2 anastomoses per person).<br>**Week 5 (10 hours):** First "6-stitches test" on a rubber glove. Chicken femoral artery anastomosis (2 anastomoses per person).<br>**End of basic training.**<br>**Advanced Microsurgical Training**<br>**Week 6 (12 hours):** Chicken femoral nerve suturing. Chicken femoral artery anastomosis (2 anastomoses per person).<br>**Week 7 (14 hours):** Introduction to end-to-side arterial anastomosis (1 anastomosis per person).<br>**Week 8 (16 hours):** Chicken femoral artery anastomosis. Introduction to vein suturing techniques. Chicken femoral vein anastomosis (2 anastomoses per person).<br>**Week 9 (18 hours):** Second "6-stitches test." Artery anastomosis with vein conduit (2 anastomoses per person).<br>**Week 10 (20 hours):** Chicken femoral nerve suturing. Artery anastomosis with vein conduit (3 anastomoses per person).<br>**Week 11 (22 hours):** Continued artery anastomosis with vein conduit (3 anastomoses per person).<br>**Week 12 (24 hours):** Third "6-stitches test." Repair of a chicken femoral nerve defect using a vein conduit (2 repairs per person).<br>**Week 13 (26 hours):** Chicken femoral artery anastomosis (test). Repair of a chicken femoral nerve defect with a vein conduit (3 repairs per person).<br>**Week 14 (28 hours):** Complete chicken femoral vascular bundle repair (artery, vein, and nerve) (3 repairs per person).<br>**Week 15 (30 hours):** Chicken femoral artery anastomosis (test). Chicken femoral artery and vein anastomosis (3 anastomoses per person). **End of advanced training.**
