# Supplementary material for: Validated Microsurgical Training Programmes: A Systematic Review of the Current Literature
Source: J Clin Med. 2025 Oct 22;14(21):7452. doi: 10.3390/jcm14217452 (PMC12609450; doi:10.3390/jcm14217452)
Supplement: Supplementary file 1 [file jcm-14-07452-s001.zip › Supplementary Table S2- MERSQI scoring details; .pdf]

No.	Paper	Study design	Sampling Institutions	Sampling Response Rate	Type of Data	Validity Evidence	Data Analysis Sophistication	Data Analysis Appropriate	Outcome	Total
1	Berretti et al (2018)	2	0.5	1.5	3	2	2	1	1.5	**13.5**
2	Bigorre et al (2020)	1.5	0.5	1.5	3	1	2	1	1.5	**12**
3	Chacon et al (2020)	1.5	0.5	1.5	3	2	2	1	1.5	**13**
4	Chauhan et al. (2023)	2	0.5	1.5	3	2	2	1	2	**14**
5	Cui et al (2024)	3	0.5	1.5	3	1	2	1	2	**14**
6	Esanu et al (2022)	3	0.5	1.5	3	3	2	1	1.5	**15.5**
7	Geoghegan et al (2023)	1.5	0.5	0.5	3	3	2	1	1.5	**13**
8	Guerreschi et al (2014)	2	0.5	1.5	3	1	2	1	1.5	**12.5**
9	Jensen et al (2023)	3	0.5	1.5	3	0	2	1	1.5	**12.5**
10	Juratli et al (2021)	1.5	0.5	1.5	3	1	1	1	1.5	**11**
11	Ko et al (2015)	1.5	0.5	1.5	3	1	2	1	1.5	**12**
12	Komatsu et al (2013)	1	0.5	1.5	3	1	1	1	1.5	**10.5**
13	Lahiri et al (2020)	2	0.5	1.5	3	1	2	1	1.5	**12.5**
14	LeHanneur et al (2024)	3	0.5	1.5	3	2	2	1	1.5	**14.5**
15	Luther et al (2019)	1.5	0.5	1.5	3	3	2	1	1.5	**14**
16	Masud et al (2017)	3	0.5	1.5	3	2	2	1	1.5	**14.5**
17	Mattar et al (2021)	1.5	0.5	0.5	3	3	2	1	1.5	**13**
18	Onoda et al (2016)	1	0.5	1.5	3	1	2	1	1.5	**11.5**
19	Perez- Abadia et al (2017)	1.5	1.5	1.5	3	1	1	1	2	**12.5**
20	Perez- Abadia et al (2023)	1	1.5	1	3	1	1	1	1.5	**11**
21	Rodriguez et al (2016)	2	0.5	1.5	3	3	2	1	1.5	**14.5**
22	Santyr et al (2022)	2	0.5	1.5	3	3	2	1	2	**15**
23	Trignano et al (2017)	3	0.5	1.5	3	1	2	1	2	**14**
24	Zambrano- Jerez et al (2024)	1.5	0.5	1.5	3	2	2	1	1.5	**13**
25	Zyluk et al (2019)	1	0.5	1.5	3	1	1	1	1.5	**10.5**
	**Mean MERSQI Score**									**12.96**

MERSQI[c]

Study design	• Single-group cross-sectional or single-group posttest only: 1<br>• Single-group pretest and posttest: 1.5<br>• Nonrandomized, 2 group: 2<br>• Randomized controlled trial: 3	• Survey studies are cross-sectional.<br>• Case–control and cohort studies (2 or more defined cohorts) are considered 2-group nonrandomized.
Sampling: institutions	• 1 institution: 0.5<br>• 2 institutions: 1<br>• 3 or more institutions: 1.5	• Number of institutions refers to origin of study participants (not study authors).
Sampling: response rate	• Not applicable<br>• < 50% or not reported: 0.5<br>• 50%–74%: 1<br>• ≥ 75%: 1.5	• Response rate is the proportion of those eligible who completed the posttest or survey. For intervention studies, this is the proportion of those enrolled who completed the intervention evaluation.<br>• Use "not applicable" only if a response rate truly does not apply (e.g., data obtained from a medical record or professional organization database).
Type of data	• Assessment by study participant: 1<br>• Objective: 3	• Observer ratings are considered objective.
Validity evidence for evaluation instrument scores	• Not applicable<br>• Content: 1<br>• Internal structure: 1<br>• Relationships to other variables: 1	• Relevant content evidence would include using theory, guidelines, experts, and existing instruments to identify or refine the instrument.<br>• Relevant internal structure evidence would include all reliability (internal consistency, interrater, interstation, and test–retest) and factor analysis.<br>• Relevant evidence of relationships to other variables would include expert–novice comparisons and concurrent or predictive correlation with other variables.<br>• Use "not applicable" only if the study does not measure a psychological construct *and* there is no instrument to rate (e.g., gender as the sole outcome); should be used very rarely.
Data analysis: sophistication	• Descriptive analysis only: 1<br>• Beyond descriptive analysis: 2	• Descriptive analyses include frequency, mean, and median.<br>• Any test of statistical inference is considered "beyond descriptive."
Data analysis: appropriate	• Data analysis appropriate for study design and type of data: 1	• Considered "no" if there is a statistical error or if authors failed to analyze data at all.
Outcome	• Satisfaction, attitudes, perceptions, opinions, general facts: 1<br>• Knowledge, skills: 1.5<br>• Behaviors: 2<br>• Patient/health care outcome: 3	• General facts include participant demographics.<br>• Knowledge/skills are in a test setting (paper, computer, simulation, or patients in a nonauthentic setting).<br>• Behaviors are physician actions with real patients in a clinical context, or other activities in a real context.<br>• Patient/health care outcomes are actual effects on real patients, programs, or society.
